# Fotobiomodulação Combinada ao Treinamento Intervalado de Intensidade Moderada ou Alta no Consumo de Oxigênio e na Tolerância ao Exercício em Pacientes com Insuficiência Cardíaca

**DOI:** 10.36660/abc.20250086

**Published:** 2025-12-08

**Authors:** Diego Busin, Leandro Tolfo Franzoni, Douglas Turella, Olga Sergueevna Tairova, Anderson Donelli da Silveira, Ricardo Stein, Gabriel Lopes Amorim, Pedro Dal Lago, Ramiro Barcos Nunes

**Affiliations:** 1 Programa de Pós-Graduação em Ciências da Reabilitação Universidade Federal de Ciências da Saúde de Porto Alegre Porto Alegre RS Brasil Programa de Pós-Graduação em Ciências da Reabilitação – Universidade Federal de Ciências da Saúde de Porto Alegre, Porto Alegre, RS – Brasil; 2 Programa de Pós-Graduação em Ciências da Saúde: Cardiologia e Ciências Cardiovasculares Universidade Federal do Rio Grande do Sul Porto Alegre RS Brasil Programa de Pós-Graduação em Ciências da Saúde: Cardiologia e Ciências Cardiovasculares – Universidade Federal do Rio Grande do Sul, Porto Alegre, RS – Brasil; 3 Programa de Reabilitação Cardíaca Centro Clínico Universidade de Caxias do Sul Caxias do Sul RS Brasil Programa de Reabilitação Cardíaca – Centro Clínico da Universidade de Caxias do Sul, Caxias do Sul, RS – Brasil

**Keywords:** Treino Aeróbico, Terapia com Luz de Baixa Intensidade, Insuficiência Cardíaca, Teste de Esforço

## Abstract

**Fundamento:**

O treinamento aeróbico (TA), seja de intensidade moderada ou alta, é amplamente utilizado na reabilitação cardiovascular. A terapia por fotobiomodulação (TFBM) tem demonstrado potencial para melhorar o desempenho físico em atletas; no entanto, seus efeitos em pacientes com insuficiência cardíaca (IC) ainda são incertos.

**Objetivo:**

Investigar se a adição da TFBM ao TA de intensidade moderada ou alta melhora o desempenho cardiorrespiratório ao exercício em indivíduos com IC.

**Métodos:**

Ensaio clínico não randomizado com 49 pacientes com IC (idade média de 62,7 anos; fração de ejeção < 40%), alocados em 5 grupos: i) treinamento moderado isolado, ii) treinamento moderado + TFBM, iii) treinamento de alta intensidade isolado, iv) treinamento de alta intensidade + TFBM, e v) grupo controle. Todos os participantes foram submetidos a uma intervenção de 10 semanas. O teste de exercício cardiorrespiratório foi utilizado para avaliar o VO_2_pico, a eficiência ventilatória e a tolerância ao exercício. A análise estatística foi realizada por meio de Equações de Estimação Generalizadas, com nível de significância de 5% (p < 0,05).

**Resultados:**

Foram observadas melhorias significativas na velocidade e na inclinação durante o teste de esforço nos grupos submetidos ao treinamento, especialmente no grupo de alta intensidade. Este grupo apresentou maior aumento no consumo de oxigênio (diferença média: 1,80 ml.kg^-1^.min^-1^ ± 0,59 ml.kg^-1^.min^-1^; p = 0,002) e melhor desempenho no tempo até a exaustão (velocidade: p = 0,05; inclinação: p < 0,01). As comparações entre os grupos revelaram que a TFBM não proporcionou efeitos adicionais relevantes quando combinada ao treinamento.

**Conclusão:**

O TA, sobretudo em alta intensidade, melhora o desempenho ao exercício em pacientes com IC. A adição da TFBM não conferiu benefícios adicionais significativos neste estudo. Novos ensaios utilizando protocolos otimizados de TFBM são recomendados.

## Graphical abstract

**Figura Central:**
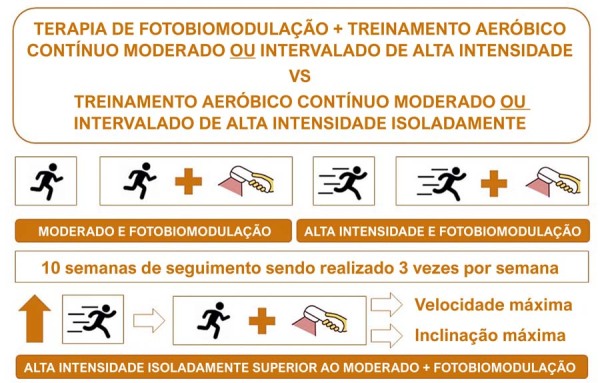
Fotobiomodulação Combinada ao Treinamento Intervalado de Intensidade Moderada ou Alta no Consumo de Oxigênio e na Tolerância ao Exercício em Pacientes com Insuficiência Cardíaca

## Introdução

A insuficiência cardíaca (IC) é considerada uma pandemia global, afetando aproximadamente 64,3 milhões de pessoas em todo o mundo.^1^ Pacientes com IC frequentemente apresentam redução da capacidade funcional, geralmente avaliada por meio do VO_2_pico.^2^ O teste de exercício cardiorrespiratório (TECR) é reconhecido como o padrão-ouro para avaliação do VO_2_pico,^3 , 4^ além de fornecer métricas importantes sobre a tolerância ao exercício, como tempo até a exaustão, velocidade e inclinação.^5^ As limitações ao exercício na IC são frequentemente atribuídas a mecanismos periféricos, em especial à capacidade comprometida de extração de oxigênio pelo músculo esquelético.^2^ Esse déficit decorre de disfunção mitocondrial, contribuindo para a fadiga precoce.^6^

O treinamento aeróbico (TA) — seja sob a forma de treinamento contínuo de intensidade moderada (TCIM)^7 , 8^ ou treinamento intervalado de alta intensidade (TIAI)^9 - 11^ — tem se mostrado eficaz na melhora do metabolismo mitocondrial. Além disso, estratégias terapêuticas direcionadas ao músculo esquelético, como a terapia por fotobiomodulação (TFBM), podem atuar como adjuvantes ao TCIM ou TIAI, ao potencializar a extração periférica de oxigênio e, consequentemente, melhorar a capacidade funcional. A TFBM tem sido proposta como um recurso para aumentar a produção de ATP e melhorar a cinética do VO_2_.

Evidências em indivíduos saudáveis indicam que a TFBM modula a cinética do VO_2_.^12^ Além disso, não foram observadas diferenças significativas entre a aplicação da TFBM antes ou após as sessões de exercício, sendo relatadas melhorias no VO_2_pico e no tempo até a exaustão após 12 semanas de uso.^12^ Uma metanálise também demonstrou efeitos positivos da TFBM sobre o desempenho muscular e a fadiga; no entanto, a qualidade metodológica dos estudos incluídos foi, em geral, baixa.^13^

No contexto das doenças cardiovasculares, a TFBM não demonstrou eficácia em melhorar agudamente a capacidade funcional de pacientes submetidos à cirurgia de revascularização do miocárdio.^14^ De modo semelhante, em indivíduos com IC, a TFBM não apresentou efeitos significativos na melhora da capacidade funcional, embora possa influenciar positivamente a percepção de esforço.^15^ Até o momento, os efeitos crônicos da TFBM em pacientes com IC têm sido explorados principalmente em modelos animais, particularmente em associação ao treinamento resistido, e não ao TA. Esses estudos apresentaram resultados promissores, com melhorias no VO_2_ e na tolerância ao exercício.^16^ Contudo, os efeitos da TFBM combinada ao TA em pacientes com IC permanecem pouco investigados, especialmente em relação às variáveis obtidas por meio do TECR.

Diante dessa lacuna na literatura, o objetivo principal deste estudo foi avaliar se a TFBM, combinada ao TCIM ou ao TIAI, oferece benefícios adicionais na elevação do VO_2_pico em pacientes com IC, em comparação ao TCIM ou TIAI isoladamente. Como objetivo secundário, buscou-se analisar os efeitos da TFBM sobre outras variáveis derivadas do TECR e sobre a tolerância ao exercício. Nossa hipótese foi de que a combinação da TFBM ao TCIM ou TIAI proporcionaria resultados superiores em comparação aos treinamentos realizados isoladamente.

## Métodos

### Desenho do estudo

Este foi um ensaio clínico não randomizado, com recrutamento por conveniência e inclusão mediante assinatura do termo de consentimento livre e esclarecido. Os participantes foram alocados em 5 grupos: grupo de TCIM, grupo de TIAI, grupo de TCIM associado à TFBM (TCIM-TFBM), grupo de TIAI associado à TFBM (TIAI-TFBM) e grupo controle (GC).

O estudo foi conduzido na Unidade de Medicina do Esporte do Centro Clínico (CECLIN) da Universidade de Caxias do Sul, entre março de 2017 e abril de 2018. Foi aprovado pelo Comitê de Ética em Pesquisa da Universidade Federal de Ciências da Saúde de Porto Alegre (número de aprovação: 6.273.174). As avaliações foram realizadas por um único examinador, cego quanto à alocação dos participantes. O estudo seguiu os princípios éticos estabelecidos nas diretrizes do *International Journal of Sports Medicine* .^17^

### Participantes

Os pacientes foram recrutados por meio do Programa de Reabilitação Cardíaca do CECLIN. Todos os participantes foram encaminhados pelo Sistema Único de Saúde, com diagnóstico clínico de insuficiência cardíaca. No momento da triagem para o programa, os pacientes foram convidados a participar do estudo. Aqueles que recusaram a participação no programa de reabilitação ainda assim foram convidados a compor o GC.

Os critérios de elegibilidade incluíram: 1) fração de ejeção < 49%; 2) idade ≥ 18 anos; 3) classe funcional II ou III da New York Heart Association; 4) estabilidade clínica por pelo menos 3 meses com tratamento medicamentoso otimizado; 5) ausência de hospitalizações nos últimos 6 meses; 6) ausência de histórico de acidente vascular cerebral isquêmico ou hemorrágico ou outras condições neurológicas que afetassem a marcha; 7) não participação em programas estruturados de exercício físico.

### Programa de intervenção

Todos os participantes encaminhados ao programa foram submetidos a uma entrevista clínica e a uma avaliação física. A alocação aos grupos de intervenção foi feita de forma sequencial (1:1:1:1), ou seja, cada novo participante era alocado a um grupo diferente do anterior, exceto no caso do GC. A intervenção teve duração de 10 semanas, com sessões realizadas 3 vezes por semana.

### Protocolos de intervenção

O protocolo de TCIM consistiu em sessões de 47 minutos, realizadas entre 70% e 75% da frequência cardíaca de reserva (FCR). O protocolo de TIAI envolveu quatro intervalos de 4 minutos entre 90% e 95% da FCR, cada um seguido de 3 minutos de recuperação ativa (caminhada leve), precedidos por aquecimento de 10 minutos a 50%-60% da FCR.

A FCR foi calculada como a diferença entre a FC máxima (obtida durante o TECR) e a frequência cardíaca de repouso (mensurada antes de cada sessão).

Os grupos com TFBM seguiram os mesmos protocolos de TCIM ou TIAI, com a adição da TFBM aplicada antes de cada sessão. O regime de exercícios foi baseado no protocolo descrito por Wisløff et al.^11^ A FC foi monitorada continuamente durante as sessões com oxímetro de pulso digital (NoninOnix 9500, Reino Unido), e a intensidade foi ajustada em tempo real para manter a frequência dentro da zona alvo.

### Protocolo de TFBM

A TFBM foi administrada com um dispositivo laser de GaAlAs (ISO:13485, modelo 2779, Chattanooga Group – Intelect® Mobile Laser, Austin, TX, EUA). A terapia com laser de baixa potência ( *low-level laser therapy* , LLLT), com comprimento de onda de 810 nm, foi aplicada bilateralmente, com contato direto e leve pressão sobre a pele.

Cada membro inferior recebeu irradiação em 6 pontos anatômicos: vasto medial, vasto lateral, bíceps femoral, semitendíneo, gastrocnêmio lateral e gastrocnêmio medial. Os parâmetros técnicos seguiram recomendações clínicas e científicas consolidadas para melhora do desempenho e da recuperação pós-exercício, conforme descrito por Leal-Junior et al.^18^ Após a aplicação da TFBM, os participantes iniciavam a sessão de TCIM ou TIAI conforme sua alocação.

### Coleta e análise de dados

#### Variáveis do TECR

O TECR foi realizado após aquecimento de 10 minutos, utilizando analisador de gases VO2000 (Medical Graphics Diagnostics Corporation, EUA) integrado à esteira Super ATL 300 (Inbramed, Porto Alegre, RS, Brasil). Foi utilizado um protocolo em rampa, com velocidade inicial de 3 km.h^-^ ^1^ sem inclinação. A cada minuto, a velocidade aumentava 0.3 km.h^-1^ e a inclinação 1,6%, até exaustão voluntária.

Os gases expirados foram coletados a cada 20 segundos para cálculo dos parâmetros ventilatórios. Durante o teste, foram monitoradas continuamente variáveis como pressão arterial, frequência cardíaca, ventilação por minuto (VE), VO_2_, VCO_2_, quociente respiratório (RER) e equivalentes ventilatórios (VE/VO_2_ e VE/VCO_2_). Esses dados foram utilizados para determinar o VO_2_pico, os limiares ventilatórios (LV1 e LV2) e outros marcadores metabólicos e ventilatórios relevantes.^19^

Também foram registradas medidas de tolerância ao exercício, como tempo até a exaustão, velocidade máxima e inclinação máxima atingida.

## Análise estatística

As variáveis categóricas foram apresentadas como frequências absolutas (n) e relativas (%), enquanto os dados contínuos foram expressos como média ± desvio padrão (DP). As comparações basais foram realizadas por meio de ANOVA de uma via, com teste post-hoc de Tukey para comparações pareadas. A normalidade foi avaliada pelo teste de Shapiro-Wilk.

Para a análise principal, aplicaram-se Equações de Estimação Generalizadas (GEE) para avaliar os efeitos de grupo, tempo e interação grupo*tempo. Quando os efeitos de interação se aproximaram da significância (p ≤ 0,10), testes post-hoc de Bonferroni foram realizados. Todos os resultados foram reportados como média ± erro padrão (EP), e as diferenças médias (DM) foram acompanhadas de intervalos de confiança de 95% (IC 95%).

O nível de significância adotado foi α = 5% (p < 0,05). As análises estatísticas foram realizadas no software IBM SPSS Statistics for Windows, versão 20 (IBM Corp., Armonk, N.Y., EUA).

## Resultados

Apenas 1 participante não completou o protocolo de intervenção por motivos pessoais (TIAI-TFBM, n = 9). A amostra final foi composta por 49 participantes, dos quais 67% eram do sexo masculino (n = 33; p = 0,32). A Tabela 1 apresenta as características basais da amostra. Observou-se diferença significativa na média de idade entre os grupos TIAI-TFBM e TCIM (p = 0,04). A média geral de idade da amostra foi de 62,73 ± 10,05 anos.

**Tabela 1:** – Dados de caracterização da amostra para os 5 grupos do estudo

Variável	TCIM (n = 10)	TIAI (n = 10)	TCIM-TFBM (n = 10)	TIAI-TFBM (n = 9)	GC (n = 10)	Valor p
**Caracterização da amostra**						
Sexo (M/F), n	8/2	7/3	4/6	7/2	7/3	0,32
Idade (anos)	68,70 ± 9,97*	63,10 ± 8,22	62,10 ± 11,42	54,55 ± 7,92*	64,50 ± 8,55	0,04
Fração de ejeção (%)	37,70 ± 7,35	38,70 ± 4,78#	33,90 ± 7,01	31,11 ± 5,60#	34,40 ± 5,37	0,04
Massa corporal (kg)	73,49 ± 15,56	77,28 ± 9,49	67,13 ± 20,16	84,44 ± 12,25	74,66 ± 16,23	0,16
IMC (kg/m^2^)	26,27 ± 3,94	28,94 ± 5,53	25,53 ± 6,38	32,03 ± 6,80	27,77 ± 5,58	0,68
**Fatores de risco**						
Dislipidemia, n (%)	4 (40)	6 (60)	7 (70)	7 (77,8)	4 (40)	0,32
Hipertensão, n (%)	7 (70)	7 (70)	9 (90)	6 (66,7)	10 (100)	0,25
Tabagismo, n (%)	4 (40)	6 (60)	3 (30)	4 (44,4)	5 (50)	0,73
Sobrepeso, n (%)	5 (50)	7 (70)	2 (20)	6 (66,7)	6 (60)	0,17
Diabetes, n (%)	1 (10)	3 (30)	2 (20)	3 (33,3)	1 (10)	0,58
Histórico familiar de DCV, n (%)	7 (70)	6 (60)	1 (10)	1 (11,1)	1 (10)	0,003
**Uso de medicamentos**						
Anticoagulante, n (%)	10 (100)	10 (100)	8 (80)	8 (88,9)	9 (90)	0,43
Vasodilatador, n (%)	6 (60)	5 (50)	7 (70)	6 (66,7)	8 (80)	0,69
IECA, n (%)	6 (60)	6 (60)	3 (30)	2 (22,2)	4 (40)	0,32
Antiarrítmico, n (%)	5 (50)	2 (20)	3 (30)	1 (11,1)	4 (40)	0,36
Hipolipemiante, n (%)	8 (80)	8 (80)	6 (60)	8 (88,9)	7 (70)	0,63
Digoxina, n (%)	3 (30)	0 (0)	1 (10)	1 (11,1)	1 (10)	0,27
Betabloqueador, n (%)	7 (70)	10 (100)	9 (90)	7 (77,8)	10 (100)	0,15
Diurético, n (%)	6 (60)	9 (90)	8 (80)	6 (66,7)	7 (70)	0,58
Hipoglicemiante, n (%)	1 (10)	4 (40)	2 (20)	2 (22,2)	2 (20)	0,60
**Condições médicas**						
CRM, n (%)	3 (30)	3 (30)	2 (20)	0 (0)	2 (20)	0,48
Valvopatia, n (%)	0 (0)	0 (0)	1 (10)	1 (11,1)	0 (0)	0,50
Angioplastia, n (%)	9 (90)	8 (80)	5 (50)	8 (88,9)	8 (80)	0,20
IAM, n (%)	7 (70)	6 (60)	6 (60)	5 (55,6)	3 (30)	0,45
DAC, n (%)	8 (80)	9 (90)	6 (60)	5 (55,6)	5 (50)	0,26
Angina, n (%)	2 (20)	1 (10)	0 (0)	0 (0)	0 (0)	0,23
Miocardiopatia dilatada, n (%)	0 (0)	4 (40)	1 (10)	2 (22,2)	4 (40)	0,12
FA, n (%)	3 (30)	0 (0)	0 (0)	0 (0)	0 (0)	0,01
CDI, n (%)	2 (20)	0 (0)	1 (10)	0 (0)	0 (0)	0,23
Aneurisma de aorta, n (%)	1 (10)	0 (0)	0 (0)	0 (0)	0 (0)	0,40
Marca-passo, n (%)	2 (20)	1 (10)	1 (10)	0 (0)	2 (20)	0,65
Arritmia, n (%)	3 (30)	0 (0)	1 (10)	0 (0)	1 (10)	0,16

CDI: cardiodesfibrilador implantável; CRM: cirurgia de revascularização miocárdica; DAC: doença arterial coronariana; DCV: doença cardiovascular; FA: fibrilação atrial; GC: grupo controle; IAM: infarto agudo do miocárdio; IECA: inibidor da enzima conversora da angiotensina; IMC: índice de massa corporal; TCIM: grupo de treinamento contínuo de intensidade moderada; TCIM-TFBM: grupo de treinamento contínuo de intensidade moderada com fotobiomodulação; TIAI: grupo de treinamento intervalado de alta intensidade; TIAI-TFBM: grupo de treinamento intervalado de alta intensidade com fotobiomodulação.

**Figura 1:**
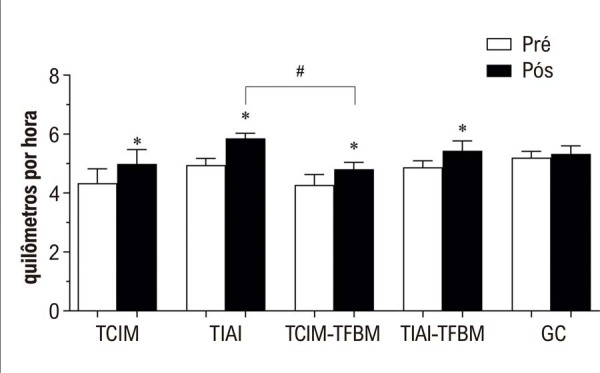
– Velocidade nos períodos pré e pós-intervenção para os 5 grupos. GC: grupo controle; TCIM: grupo de treinamento contínuo de intensidade moderada; TCIM-TFBM: grupo de treinamento contínuo de intensidade moderada com fotobiomodulação; TIAI: grupo de treinamento intervalado de alta intensidade; TIAI-TFBM: grupo de treinamento intervalado de alta intensidade com fotobiomodulação. *indica diferenças significativas entre os períodos pré e pós-intervenção dentro do mesmo grupo. # indica diferenças significativas entre grupos no mesmo ponto temporal.

**Figura 2:**
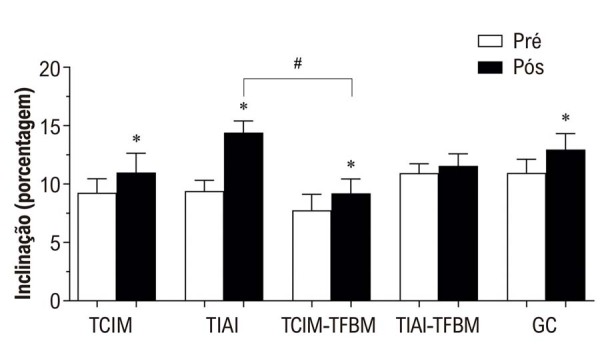
– Inclinação nos períodos pré e pós-intervenção para os 5 grupos. GC: grupo controle; TCIM: grupo de treinamento contínuo de intensidade moderada; TCIM-TFBM: grupo de TCIM associado à terapia por fotobiomodulação; TIAI: grupo de treinamento intervalado de alta intensidade; TIAI-TFBM: grupo de TIAI associado à terapia por fotobiomodulação. *indica diferenças significativas entre os períodos pré e pós-intervenção dentro do mesmo grupo. # indica diferenças significativas entre grupos no mesmo ponto temporal.

Os valores médios das variáveis do TECR e de tolerância ao exercício para todos os grupos estão apresentados na Tabela Suplementar 1 . Os valores de p referentes ao teste de efeitos do modelo GEE estão disponíveis na Tabela Suplementar 2 . Os principais resultados com diferenças estatisticamente significativas estão ilustrados na Figura 1 (velocidade) e na Figura 2 (inclinação). A Figura Central também resume os achados principais e descreve a estrutura geral do protocolo de intervenção.

Embora tenham sido observadas melhorias numéricas em variáveis como VO_2_pico, declive VE/VCO_2_ e declive da eficiência de captação de oxigênio, não foram encontradas diferenças estatisticamente significativas entre os grupos para essas variáveis. Esses achados sugerem que a intervenção, com ou sem a adição da fotobiomodulação, não produziu alterações relevantes nesses parâmetros do TECR.

## Discussão

Até onde sabemos, este é o primeiro estudo a investigar a efetividade da TFBM quando combinada ao TCIM ou ao TIAI, bem como a avaliar se sua associação oferece benefícios adicionais em relação ao TCIM ou TIAI isoladamente na melhora do VO_2_pico, de outras variáveis derivadas do TECR e da tolerância ao exercício em indivíduos com IC. Os principais achados estão resumidos a seguir.

O principal resultado foi que todos os grupos de intervenção apresentaram melhorias significativas na tolerância ao exercício, evidenciadas pelo aumento da velocidade e da inclinação durante o TECR. No entanto, ao comparar os grupos que receberam TFBM com aqueles que não a receberam, não foram observados benefícios adicionais da TFBM em nenhuma das variáveis analisadas. Essa constatação foi corroborada pela análise post-hoc, que não revelou diferenças significativas entre os grupos com e sem TFBM.

Nossa hipótese inicial era de que a TFBM, combinada ao TCIM ou ao TIAI, proporcionaria resultados superiores em comparação ao uso isolado dessas modalidades. Essa suposição baseava-se no racional mecanístico de que a TFBM melhora a atividade mitocondrial ao promover a absorção de energia luminosa por cromóforos específicos, estimulando vias de sinalização intracelular e aumentando a produção de ATP. Esperava-se que esses mecanismos amplificassem as adaptações fisiológicas induzidas pelo TA.^20^ No entanto, nossos resultados não sustentaram essa hipótese, visto que as melhorias nos desfechos relacionados à tolerância ao exercício foram semelhantes entre todos os grupos de intervenção, independentemente do uso da TFBM. Ainda assim, os benefícios observados ressaltam o valor clínico do TA — especialmente do TIAI — no manejo da IC.

Estudos prévios com indivíduos saudáveis demonstraram que a TFBM combinada ao TCIM pode levar a maiores melhorias no VO_2_pico e no tempo até a exaustão em comparação ao TCIM isolado.^12^ Esses achados contrastam com os nossos e podem ser explicados por diferenças metodológicas e populacionais. Por exemplo, Miranda et al. utilizaram uma combinação de laser, LED infravermelho e LED vermelho, com dose total de energia substancialmente maior (2040 J) e 17 pontos de irradiação, enquanto nosso estudo empregou apenas laser, com dose inferior (360 J) e 6 pontos de irradiação.^12^ Além disso, enquanto Miranda et al. estudaram indivíduos saudáveis submetidos apenas ao TCIM, nosso estudo incluiu pacientes com IC e protocolos tanto de TCIM quanto de TIAI.^12^

Em populações saudáveis, a TFBM tem demonstrado efeitos positivos sobre desempenho e fadiga, especialmente quando empregada com comprimentos de onda de 655/950 nm (60/300 J) e potência máxima de 200 mW por diodo, aplicada antes do exercício.^13^ Embora alguns desses parâmetros sejam semelhantes aos utilizados em nosso estudo, a literatura permanece heterogênea e a qualidade geral das evidências é baixa, conforme demonstrado em metanálise.^12^ Alguns estudos sugerem que doses mais altas de LLLT podem ser mais eficazes, o que reforça a necessidade de novos ensaios — especialmente em populações clínicas como pacientes com IC, onde a evidência ainda é limitada.

Estudos pré-clínicos demonstraram efeitos favoráveis do LLLT em modelos animais de IC,^16 , 21^ e, em indivíduos saudáveis, o LLLT tem sido associado a melhorias na capacidade funcional e em indicadores de desempenho.^22 - 25^ No entanto, em populações com doenças cardiovasculares, incluindo IC, os estudos não evidenciaram melhorias agudas na capacidade funcional quando o LLLT foi aplicado antes das avaliações.^14 , 15^ No presente estudo, a TFBM foi aplicada antes de cada sessão de exercício ao longo de 10 semanas, mas não foram observados efeitos adicionais, sugerindo que o volume acumulado de aplicações, por si só, pode não ser um fator determinante nos desfechos.

Os parâmetros obtidos pelo TECR fornecem valor diagnóstico e prognóstico em pacientes com IC, além de oferecerem informações essenciais sobre a tolerância ao exercício.^26^ Nesse contexto, nossos resultados indicam que o TA — particularmente o TIAI sem TFBM — foi mais eficaz na melhora de parâmetros de tolerância ao exercício, como velocidade e inclinação na esteira, em comparação ao TCIM associado à TFBM. Esses achados estão em consonância com estudos prévios que relataram benefícios significativos do TIAI isolado sobre a aptidão cardiorrespiratória e a tolerância ao exercício em pacientes com IC.^2 , 9 - 11 , 26^

Embora não tenhamos observado diferenças estatisticamente significativas no VO_2_pico entre os grupos, os aumentos absolutos observados em todos os grupos de intervenção ainda são clinicamente relevantes. As melhorias no VO_2_pico relatadas neste estudo foram menores do que os 6 ml.kg^-1^.min^-1^ descritos por Wisløff et al.^11^ No entanto, o grupo TIAI-TFBM apresentou melhora de 13,5%, superior aos 11,2% relatados para o TIAI isolado no estudo de Ulbrich et al.^27^ Ademais, os aumentos de VO_2_pico nos grupos TIAI e TIAI-TFBM superaram o limiar de 1 ml.kg^-1^.min^-1^, considerado clinicamente significativo para a manutenção da autonomia e da capacidade funcional nas atividades diárias.^28^

Apesar da ausência de diferenças significativas no VO_2_pico entre os grupos, os ganhos observados em métricas de desempenho, como velocidade e inclinação, sugerem possíveis adaptações relacionadas à economia de movimento. Ou seja, os participantes podem ter se tornado mais eficientes em seus movimentos, realizando tarefas de maior intensidade com igual ou menor custo metabólico. Esse tipo de adaptação é particularmente relevante em pacientes com IC, por refletir uma melhora funcional sem sobrecarga fisiológica adicional.

### Forças e limitações do estudo

O presente estudo apresenta diversas fortalezas. Seu delineamento é aplicável à prática clínica e gerou resultados confiáveis sobre a capacidade funcional com base em métricas derivadas do TECR — considerado o padrão-ouro para avaliação da aptidão cardiorrespiratória. Até onde sabemos, este é o primeiro estudo a examinar os efeitos da TFBM combinada a duas modalidades distintas de TA — o TCIM e o TIAI — em pacientes com IC. Ademais, trata-se, aparentemente, do primeiro estudo a empregar TFBM de forma prolongada como parte de um programa de reabilitação cardíaca nessa população. Esses achados inéditos contribuem para o crescente corpo de evidências que apoiam a TFBM como uma possível terapia não farmacológica adjuvante no manejo da IC, demonstrando que seu uso é tão seguro e viável quanto o TA convencional isolado.

No entanto, este estudo apresenta limitações importantes. Primeiramente, não foi incluído um grupo tratado exclusivamente com TFBM, o que teria permitido isolar seus efeitos. Em segundo lugar, o tamanho amostral pode ter sido insuficiente para detectar diferenças significativas em algumas comparações pré e pós-intervenção. A ausência de um grupo placebo submetido aos mesmos protocolos de TA, mas sem a irradiação ativa da TFBM, constitui outra limitação, assim como o delineamento não randomizado do estudo. Adicionalmente, este ensaio clínico não foi registrado em base pública, o que limita sua transparência e reprodutibilidade. Por fim, os participantes do GC foram aqueles que recusaram participar do programa de intervenção, o que introduz um viés de seleção que deve ser reconhecido.

## Conclusão

Em síntese, nossos achados indicam que a TFBM, quando combinada ao TCIM ou ao TIAI, não demonstrou eficácia superior na melhora dos desfechos avaliados em comparação ao TCIM ou TIAI isoladamente. Embora a TFBM tenha demonstrado benefícios potenciais em outros contextos, seu valor adicional na melhora da tolerância ao exercício, do VO_2_pico e de outras variáveis derivadas do TECR em pacientes com IC não foi confirmado neste estudo.

Com base no racional mecanístico da TFBM — relacionado ao estímulo do metabolismo celular e ao aumento da produção de ATP —, recomendamos que estudos futuros explorem parâmetros terapêuticos alternativos, como doses energéticas mais elevadas, maior número de pontos de irradiação ou diferentes comprimentos de onda, a fim de avaliar de forma mais aprofundada o potencial da TFBM como intervenção não farmacológica na IC. Pesquisas futuras poderão oferecer contribuições valiosas para a otimização de terapias adjuvantes nesta população.

## *Material suplementar

Material suplementar 1 Para informação adicional do Material Suplementar 1, por favor, clique aqui. https://abccardiol.org/supplementary-material/2025/12211/2025-0086_AO_Supplementary_table_1.pdf

Material suplementar 2 Para informação adicional do Material Suplementar 2, por favor, clique aqui. https://abccardiol.org/supplementary-material/2025/12211/2025-0086_AO_Supplementary_table_2.pdf
