# Supplementary material for: Fotobiomodulação Combinada ao Treinamento Intervalado de Intensidade Moderada ou Alta no Consumo de Oxigênio e na Tolerância ao Exercício em Pacientes com Insuficiência Cardíaca
Source: Arq Bras Cardiol. 2025 Dec 8;122(11):e20250086. [Article in Portuguese] doi: 10.36660/abc.20250086 (PMC12711225; doi:10.36660/abc.20250086)
Supplement: Material suplementar 1 [file 0066-782x-abc-122-11-e20250086-suppl01.pdf]

Supplementary table 2. Generalizing Estimating Equations model effects.

Variable	Group	Time	Group*Time
**VO_2peak (ml.kg.$^{-1}$min.$^{-1}$)**	0.30	<0.01	0.22
**VO_2 1st VT (ml.kg.$^{-1}$min.$^{-1}$)**	0.16	0.10	0.10
**VO_2 2nd VT (ml.kg.$^{-1}$min.$^{-1}$)**	0.14	<0.01	0.89
**HR peak (bpm)**	0.85	0.11	0.17
**HR 1st VT (bpm)**	0.89	0.68	0.15
**HR 2nd VT (bpm)**	0.82	0.16	0.87
**VE/VCO2 at peak**	0.04	0.34	0.46
**VE/VCO2slope**	<0.01	0.48	0.87
**OUES**	0.16	0.93	0.30
**O2 Pulse**	0.41	0.02	0.79
**Total Time Test**	0.15	0.01	0.21
**Time Test 1st VT**	0.08	<0.01	0.32
**Time Test 2nd VT**	0.03	<0.01	0.35
**Speed Test**	0.05	<0.01	0.05
**Speed 1st VT**	0.01	<0.01	0.15
**Speed 2nd VT**	0.31	<0.01	0.44
**Total Time Test**	0.17	<0.01	<0.01
**Time Test 1st VT**	0.01	<0.01	0.15
**Time Test 2nd VT**	0.05	<0.01	0.21

Notes: VT – ventilatory threshold; OUES – oxygen uptake efficiency slope.
