# Supplementary material for: Fotobiomodulação Combinada ao Treinamento Intervalado de Intensidade Moderada ou Alta no Consumo de Oxigênio e na Tolerância ao Exercício em Pacientes com Insuficiência Cardíaca
Source: Arq Bras Cardiol. 2025 Dec 8;122(11):e20250086. [Article in Portuguese] doi: 10.36660/abc.20250086 (PMC12711225; doi:10.36660/abc.20250086)
Supplement: Material suplementar 2 [file 0066-782x-abc-122-11-e20250086-suppl02.pdf]

Supplementary table 1. Outcomes in pre- and post-intervention comparisons between the five groups.

	CMTG (n=10)		HIITG (n=10)		CMT-PBMTG (n=10)		HIIT-PBMTG (n=9)		CG (n=10)	
**Variable**	**Pre**	**Post**	**Pre**	**Post**	**Pre**	**Post**	**Pre**	**Post**	**Pre**	**Post**
**VO_2peak ($ml.kg.^{-1}min.^{-1}$)**	16.2 ± 1.7 (12,7; 19,6)	17.2 ± 1.7 (13,7; 20,7)	18.5 ± 0.8 (16,9; 20,1)	19.7 ± 0.9 (17,9; 21,6)	16.4 ± 1.3 (13,7; 19,1)	17 ± 1.1 (14,7; 19,4)	16 ± 1.4 (13,1; 18,9)	18.5 ± 1.3 (15,9; 21,1)	19.2 ± 1.3 (16,6; 21,9)	19.5 ± 1.4 (16,7; 22,3)
**VO_2 1st VT ($ml.kg.^{-1}min.^{-1}$)**	11.1 ± 1.2 (8,7; 13,6)	11.1 ± 1.1 (9; 13,3)	13.3 ± 0.6 (11,9; 14,6)	14.2 ± 0.6 (12,9; 15,4)	11.4 ± 1.3 (8,7; 14,2)	12.2 ± 1.3 (9,5; 14,8)	13.4 ± 1.4 (10,6; 16,2)	12.9 ± 0.5 (11,8; 14)	12.5 ± 1.1 (10,2; 14,7)	13.5 ± 1 (11,5; 15,6)
**VO_2 2st VT ($ml.kg.^{-1}min.^{-1}$)**	14.5 ± 1.8 (10,9; 18,1)	14.9 ± 1.4 (12; 17,8)	15.8 ± 0.6 (14,5; 17,2)	17.1 ± 0.7 (15,6; 18,6)	12.6 ± 1.3 (9,9; 15,3)	14.3 ± 1.2 (11,9; 16,8)	15 ± 1.3 (12,4; 17,6)	16.6 ± 0.9 (14,8; 18,4)	16.6 ± 1.4 (13,8; 19,5)	18 ± 1.3 (15,4; 20,6)
**Peak HR (bpm)**	115.9 ± 9.2 (97,7; 134)	113 ± 7.2 (98,7; 127,2)	119.3 ± 3.2 (112,9; 125,6)	129 ± 4.8 (119,5; 138,4)	119 ± 6.7 (105,7; 132,2)	125.5 ± 5.4 (114,8; 136,1)	126 ± 8.4 (109,4; 142,6)	123.2 ± 4.1 (115,1; 131,3)	120.7 ± 5.2 (110,3; 131)	127.1 ± 7.2 (112,8; 141,3)
**FC 1st VT (bpm)**	94.8 ± 6.4 (82; 107,5)	97.9 ± 6.2 (85,7; 110,1)	98.2 ± 4.7 (88,8; 107,5)	93.7 ± 4.2 (85,4; 101,9)	91.5 ± 6.6 (78,3; 104,6)	101.2 ± 6.1 (89,1; 113,2)	105 ± 7 (91,2; 118,7)	97.1 ± 5.5 (86,1; 108)	98.6 ± 3.8 (98; 106,1)	102.9 ± 6 (91; 114,7)
**FC 2nd VT (bpm)**	105.5 ± 6 (93,6; 117,4)	108 ± 6.6 (95; 120,9)	107.6 ± 4.3 (99; 116,1)	111.7 ± 4.8 (102,2; 121,1)	111 ± 5.9 (99,4; 122,5)	115.6 ± 5.2 (105,3; 125,8)	114.7 ± 4.9 (105,1; 124,3)	112.8 ± 4.1 (104,6; 121)	107.3 ± 4.8 (97,7; 116,8)	113.1 ± 6.5 (100,2; 125,9)
**VE/VCO_2 at peak**	30.1 ± 1.9 (26,3; 33,9)	29.8 ± 1.7 (26,4; 33,2)	25.1 ± 0.8 (23,5; 26,8)	26.1 ± 1 (24; 28,2)	28.8 ± 1.2 (26,3; 31,3)	28.7 ± 1.9 (24,9; 32,4)	31 ± 2.3 (26,4; 35,5)	29.5 ± 1.9 (25,7; 33,3)	28.9 ± 2.1 (24,7; 33,1)	26.9 ± 1.1 (24,6; 29,2)
**VE/VCO_2 slope**	26.6 ± 2.6 (21,3; 31,8)	23.1 ± 1.3 (20,4; 25,8)	21.9 ± 0.9 (20; 23,8)	21.7 ± 1.5 (18,6; 24,8)	23.6 ± 1.6 (20,5; 26,8)	23.5 ± 2.2 (19,1; 27,9)	28 ± 3.5 (20,9: 35)	26.3 ± 1.3 (23,7; 28,8)	33.1 ± 4.4 (24,4; 41,8)	33.4 ± 5.3 (23; 43,8)
**OUES**	1.5 ± 0.1 (1,2; 1,8)	1.8 ± 0.1 (1,5; 2)	2.1 ± 0.1 (1,8; 2,5)	1.9 ± 0.2 (1,4; 2,4)	1.7 ± 0.1 (1,4; 2,1)	1.7 ± 0.1 (1,5; 1,9)	1.8 ± 0.2 (1,3; 2,3)	2.1 ± 0.2 (1,6; 2,7)	2.2 ± 0.3 (1,6; 2,9)	1.9 ± 0.2 (1,5; 2,4)

	CMTG (n=10)		HIITG (n=10)		CMT-PBMTG (n=10)		HIIT-PBMTG (n=9)		CG (n=10)	
**Variable**	**Pre**	**Post**	**Pre**	**Post**	**Pre**	**Post**	**Pre**	**Post**	**Pre**	**Post**
**O2 Pulse**	13.8 ± 1.4 (10,9; 16,7)	15 ± 1.4 (12,1; 18)	15.2 ± 0.8 (13,5; 16,9)	16.2 ± 0.8 (14,6; 17,8)	13.4 ± 1 (11,4; 15,3)	14 ± 0.6 (12,7; 15,3)	13.1 ± 1.5 (10,1; 16,2)	15.3 ± 1 (13,2; 17,3)	14.2 ± 1 (12,2; 16,2)	14.6 ± 0.5 (13,5; 15,8)
**Total Time Test**	402.2 ± 51.9 (300,4; 503,9)	391.2 ± 42.1 (308,5; 473,8)	358.8 ± 27.1 (305,5; 412,0)	499.9 ± 53.5 (394,9; 604,8)	332 ± 27.7 (277,6; 386,3)	340.4 ± 29.1 (283,3; 397,4)	357.7 ± 34.9 (289,2; 426,3)	400.7 ± 41.3 (319,6; 481,8)	364.6 ± 36.2 (293,5; 435,6)	403.2 ± 35.1 (334,3; 472)
**Time Test 1st VT**	136.1 ± 23 (90,9; 181,2)	195.4 ± 18.9 (158,2; 232,5)	142.5 ± 15.1 (112,8; 172,1)	252.6 ± 24.1 (205,2; 300)	163.6 ± 22.3 (119,7; 207,4)	224.1 ± 26.6 (171,8; 276,3)	214.2 ± 20.1 (174,7; 253,7)	250.5 ± 26.6 (198,3; 302,7)	182.4 ± 24.2 (134,8; 229,9)	220.3 ± 23 years (175,1; 265,4)
**Time Test 2nd VT**	298.2 ± 47.9 (204,1; 392,2)	333.4 ± 32.1 (270,3; 396,4)	254.2 ± 22.3 (210,4; 297,9)	421.4 ± 52.5 (318,4; 524,3)	224.6 ± 20.5 (184,3; 264,8)	287.1 ± 26.5 (235,1; 339)	293 ± 30.8 (232,4; 353,5)	374.2 ± 23.7 (327,7; 420,7)	331.3 ± 29.5 (273,3; 389,2)	380.2 ± 25.8 (329,6; 430,7)
**Speed Test**	4.3 ± 0.4 (3,4; 5,2)	4.9 ± 0.4 (4; 5,8) *	4.9 ± 0.2 (4,5; 5,3)	5.8 ± 0.1 (5,5; 6,1) * †	4.2 ± 0.3 (3,6; 4,9)	4.8 ± 0.2 (4,3; 5,2) * †	4.8 ± 0.2 (4,4; 5,5)	5.4 ± 0.3 (4,8; 6) *	5.2 ± 0.2 (4,8; 5,5)	5.3 ± 0.2 (4,8; 5,8)
**Speed 1st VT**	2.8 ± 0.2 (2,3; 3,3)	3.7 ± 0.2 (3,1; 4,2)	3.5 ± 0.1 (3,2; 3,8)	4.2 ± 0.1 (3,9; 4,5)	2.8 ± 0.3 (2; 3,5)	4 ± 0.2 (3,4; 4,5)	3.9 ± 0.1 (3,7; 4,2)	4.3 ± 0.1 (3,9; 4,6)	3.4 ± 0.2 (2,9; 3,9)	4.2 ± 0.2 (3,7; 4,6)
**Speed 2nd VT**	3.9 ± 0.4 (3; 4,7)	4.7 ± 0.4 (3,9; 5,5)	4.4 ± 0.2 (3,9; 4,9)	5.4 ± 0.1 (5; 5,8)	3.7 ± 0.3 (3,1; 4,4)	4.7 ± 0.4 (3,9; 5,5)	4.4 ± 0.2 (3,9; 4,9)	5.1 ± 0.1 (4,7; 5,4)	4.6 ± 0.1 (4,2; 5)	5.1 ± 0.2 (4,6; 5,7)
**Grade**	9.2 ± 1.1 (7; 11,4)	11 ± 1.5 (7,9; 14) *	9.4 ± 0.8 (7,6; 11,1)	14.4 ± 0.9 (12:5; 16:2) * †	7.7 ± 1.3 (5,1; 10,3)	9.2 ± 1.1 (6:8; 11:5) * †	10.9 ± 0.7 (9,4; 12,4)	11.5 ± 0.9 (9,6; 13,4)	10.9 ± 1.1 (8,7; 13,1)	12.9 ± 1.3 (10,3; 15,5) *
**Grade 1st VT**	3.4 ± 0.4 (2,4; 4,3)	5.2 ± 0.8 (3,6; 6,7)	4.3 ± 0.4 (3,3; 5,3)	6.8 ± 0.4 (6; 7,6)	4.1 ± 0.6 (2,7; 5,4)	5 ± 0.8 (3,4; 6,6)	7.1 ± 0.6 (5,8; 8,4)	6.9 ± 0.9 (5; 8,8)	4.8 ± 0.7 (3,4; 6,2)	7.3 ± 1 (5,3; 9,2)
**Grade 2nd VT**	6.8 ± 0.9 (5; 8,6)	8.7 ± 1.3 (6,1; 11,3)	7.3 ± 0.7 (5,8; 8,8)	11.3 ± 0.6 (10; 12,5)	6 ± 0.9 (4,2; 7,8)	7.4 ± 0.9 (5,5; 9,2)	9.3 ± 0.7 (7,8; 10,8)	9.8 ± 0.9 (8; 11,7)	8.7 ± 1 (6,7; 10,6)	10.3 ± 1.1 (7,9; 12,6)

Notes: CMTG – continuous moderate-intensity group; HIITG – high-intensity interval training group; CMT-PBMTG – continuous moderate-intensity group plus photobiomodulation; HIIT-PBMTG – high-intensity interval training group plus photobiomodulation; VT – ventilatory threshold; OUES - oxygen uptake efficiency slope; * indicates difference between pre- and post-moment for the same group (≤ 0.05); † indicates difference between groups for post-moment (≤ 0.05).
