# Supplementary material for: Fotobiomodulação Combinada ao Treinamento Intervalado de Intensidade Moderada ou Alta no Consumo de Oxigênio e na Tolerância ao Exercício em Pacientes com Insuficiência Cardíaca
Source: Arq Bras Cardiol. 2025 Dec 8;122(11):e20250086. [Article in Portuguese] doi: 10.36660/abc.20250086 (PMC12711225; doi:10.36660/abc.20250086)
Supplement: Translation

# Translation: Photobiomodulation Combined with Moderate or High-Intensity Interval Training on Oxygen Consumption and Exercise Tolerance in Patients with Heart Failure

**DOI:** 10.36660/abc.20250086i

**Authors:** Diego Busin, Leandro Tolfo Franzoni, Douglas Turella, Olga Sergueevna Tairova, Anderson Donelli da Silveira, Ricardo Stein, Gabriel Lopes Amorim, Pedro Dal Lago, Ramiro Barcos Nunes

**Affiliations:** 1 Brazil Programa de Pós-Graduação em Ciências da Reabilitação – Universidade Federal de Ciências da Saúde de Porto Alegre, Porto Alegre, RS – Brazil; 2 Brazil Programa de Pós-Graduação em Ciências da Saúde: Cardiologia e Ciências Cardiovasculares – Universidade Federal do Rio Grande do Sul, Porto Alegre, RS – Brazil; 3 Brazil Programa de Reabilitação Cardíaca – Centro Clínico da Universidade de Caxias do Sul, Caxias do Sul, RS – Brazil

**Keywords:** Endurance Training, Low-Level Light Therapy, Insuficiência Cardíaca, Exercise Test

## Abstract

**Background:**

Aerobic training (AT), whether moderate or high intensity, is widely employed in cardiovascular rehabilitation. Photobiomodulation therapy (PBMT) has shown potential to enhance exercise performance in athletes; however, its effects in patients with heart failure (HF) remain uncertain.

**Objective:**

To investigate whether the addition of PBMT to moderate- or high-intensity AT improves cardiopulmonary exercise performance in individuals with HF.

**Methods:**

This nonrandomized clinical trial included 49 patients with HF (mean age 62.7 years; ejection fraction < 40%), allocated into 5 groups: i) moderate training alone, ii) moderate training + PBMT, iii) high-intensity training alone, iv) high-intensity training + PBMT, and iv) control group. All participants underwent a 10-week intervention. Cardiopulmonary exercise testing was used to assess VO_2_peak, ventilatory efficiency, and exercise tolerance. Statistical analysis was performed using Generalized Estimating Equations, with alpha set at 5% (p < 0.05).

**Results:**

Significant improvements in speed and grade during the exercise test were observed in the training groups, particularly in the high-intensity training group. This group demonstrated a greater increase in oxygen consumption (mean difference: 1.80 ml.kg^-1^.min^-1^± 0.59 ml.kg^-1^.min^-1^; p = 0.002) and superior performance in time to exhaustion (speed: p = 0.05; grade: p < 0.01). Comparisons across groups revealed that PBMT did not provide any relevant additional effects when combined with training.

**Conclusion:**

AT, especially at high intensity, enhances exercise performance in patients with HF. The addition of PBMT did not confer significant additional benefits in this study. Further trials using optimized PBMT protocols are warranted.

## Introduction

Heart failure (HF) is considered a global pandemic, affecting an estimated 64.3 million individuals worldwide.^1^Patients with HF often experience a decline in functional capacity, typically assessed through VO_2_peak.^2^The cardiopulmonary exercise test (CPET) is recognized as the gold standard for evaluating VO_2_peak,^3 , 4^while also providing key metrics on exercise tolerance, including time to exhaustion, speed, and grade.^5^Exercise limitations in HF are frequently attributed to peripheral mechanisms, particularly the impaired ability to extract oxygen from skeletal muscle.^2^This deficit results from mitochondrial dysfunction, which contributes to early fatigue.^6^

Aerobic training (AT) — either as continuous moderate training (CMT)^7 , 8^or high-intensity interval training (HIIT)^9 - 11^— has proven effective in enhancing mitochondrial metabolism. In addition, therapeutic strategies targeting skeletal muscle, such as photobiomodulation therapy (PBMT), may serve as adjuncts to CMT or HIIT by further improving peripheral oxygen extraction and potentially enhancing functional capacity. PBMT has been proposed to increase the production of ATP and improve VO_2_kinetics efficiency.

Evidence in healthy individuals suggests that PBMT modulates VO_2_kinetics.^12^Furthermore, no significant differences have been observed between PBMT application before or after exercise sessions, with improvements reported in VO_2_peak and time to exhaustion following 12 weeks of use.^12^A meta-analysis has also shown favorable effects of PBMT on muscle performance and fatigue; however, the methodological quality of the included studies was generally low.^13^

In the context of cardiovascular diseases, PBMT has not demonstrated efficacy in acutely improving functional capacity in patients undergoing myocardial revascularization surgery.^14^Similarly, in individuals with HF, PBMT has not shown improvements in functional capacity, although it may positively influence perceived exertion.^15^To date, chronic PBMT effects in HF have primarily been explored in animal models, particularly in combination with resistance training rather than AT. These studies have shown encouraging results, including improvements in VO_2_and exercise tolerance.^16^Nevertheless, the effects of PBMT combined with AT in patients with HF remain underexplored, particularly regarding CPET-derived parameters.

Because of this gap in the literature, the primary aim of this study was to evaluate whether PBMT combined with CMT or HIIT offers additional benefits in increasing VO_2_peak in patients with HF, compared to CMT or HIIT alone. A secondary aim was to assess the effects of PBMT on other CPET-derived variables and exercise tolerance. We hypothesized that combining PBMT with CMT or HIIT would lead to superior outcomes across the assessed parameters compared to CMT or HIIT alone.

## Methods

### Study design

This was a nonrandomized clinical trial in which participants were recruited by convenience sampling and enrolled upon signing an informed consent form. Participants were allocated into 5 groups: CMT group (CMTG), HIIT group (HIITG), CMT combined with PBMT group (CMT-PBMTG), HIIT combined with PBMT group (HIIT-PBMTG), and a control group (CG).

The study was conducted at the Sports Medicine Unit of the Clinical Center (CECLIN) of the Universidade de Caxias do Sul between March 2017 and April 2018. It was approved by the Research Ethics Committee of the Universidade Federal de Ciências da Saúde de Porto Alegre (approval number: 6.273.174). Assessments were performed by a single evaluator who was blinded to the group assignments. The study followed the ethical principles outlined in the *International Journal of Sports Medicine* guidelines.^17^

### Participants

Patients were recruited through the Cardiac Rehabilitation Program at CECLIN. All participants were referred via the Brazilian Unified Health System with a clinical diagnosis of HF. Upon referral to the program, patients were invited to participate in the study. Individuals who declined participation in the rehabilitation program were still invited to join the CG.

Eligibility criteria included: 1) ejection fraction < 49%; 2) age ≥ 18 years; 3) New York Heart Association functional class II or III; 4) clinical stability for at least 3 months with optimized medical therapy; 5) no hospitalizations within the past 6 months; 6) no history of ischemic or hemorrhagic stroke or other neurological conditions affecting gait; 7) no participation in structured exercise programs.

### Intervention program

All participants referred to the program underwent a clinical interview followed by a physical assessment. They were allocated to one of the intervention groups using a sequential 1:1:1:1 allocation strategy — each new participant was assigned to a different group than the previous, with the exception of the CG. The intervention lasted for 10 weeks and was performed 3 times per week.

### Intervention protocols

The CMT protocol consisted of 47-minute sessions at 70%-75% of heart rate reserve (HRR). The HIIT protocol involved four 4-minute intervals at 90%-95% of HRR, each followed by a 3-minute active rest period (light walking), preceded by a 10-minute warm-up at 50%-60% of HRR.

HRR was calculated as the difference between the maximum HR (obtained during the CPET) and the resting HR (measured before each session).

The PBMT groups followed the same CMT or HIIT training protocols, with the addition of PBMT applied prior to each session. The exercise regimen was based on the protocol described by Wisloff et al.^11^HR was monitored continuously during sessions using a finger pulse oximeter (NoninOnix 9500, UK), and intensity was adjusted in real time to maintain the target HR range.

### PBMT protocol

PBMT was administered using a GaAlAs laser device (ISO:13485, model 2779, Chattanooga Group – Intelect^®^Mobile Laser, Austin, TX, USA). Low-level laser therapy (LLLT) at a wavelength of 810 nm was applied bilaterally with direct contact and light pressure to the skin.

Each lower limb received irradiation at 6 anatomical sites: vastus medialis, vastus lateralis, biceps femoris, semitendinosus, lateral gastrocnemius, and medial gastrocnemius. The technical parameters followed established clinical and scientific recommendations for enhancing performance and post-exercise recovery, as described by Leal-Junior et al.^18^After PBMT application, participants proceeded with their assigned CMT or HIIT session.

### Data collection and analysis

#### CPET variables

CPET was performed after a 10-minute warm-up using a VO2000 gas analyzer (Medical Graphics Diagnostics Corporation, USA) integrated with a Super ATL 300 treadmill (Inbramed, Porto Alegre, RS, Brazil). A ramp protocol was employed, starting at 3 km.h^-1^without grade. Speed increased by 0.3 km.h^-1^and grade by 1.6% every minute, continuing until voluntary exhaustion.

Expired gases were collected every 20 seconds to calculate ventilatory parameters. During the test, variables such as blood pressure, HR, minute ventilation (VE), VO_2_, VCO_2_, respiratory exchange ratio (RER), and ventilatory equivalents (VE/VO_2_and VE/VCO_2_) were continuously monitored. These data were used to determine VO_2_peak, ventilatory thresholds (VT1 and VT2), and other relevant metabolic and ventilatory markers.^19^

Exercise tolerance measures, including time to exhaustion, maximum speed, and maximum grade achieved, were also recorded.

## Statistical analysis

Categorical variables were presented as absolute (n) and relative (%) frequencies, while continuous data were expressed as mean ± standard deviation (SD). Baseline comparisons were conducted using one-way ANOVA with Tukey’s post-hoc test for pairwise comparisons. The Shapiro-Wilk test was used to assess normality.

For the main analysis, Generalized Estimating Equations (GEE) were applied to assess group, time, and group*time interaction effects. When interaction effects approached significance (p ≤ 0.10), Bonferroni post-hoc tests were performed. All results were reported as mean ± standard error (SE), and mean differences (MD) with 95% CIs were calculated.

Significance level was set at α = 5% (p < 0.05). Statistical analyses were performed using IBM SPSS Statistics for Windows, version 20 (IBM Corp., Armonk, N.Y., USA).

## Results

Only 1 participant did not complete the intervention protocol due to personal reasons (HIIT-PBMTG, n = 9). The final sample consisted of 49 participants, of whom 67% were men (n = 33; p = 0.32). Table 1 shows the baseline characteristics of the sample. A significant difference in mean age was observed between the HIIT-PBMTG and CMTG groups (p = 0.04). The overall mean age of the sample was 62.73 ± 10.05 years.

**Table 1:** – Sample characterization data for the 5 study groups

Variable	CMTG (n = 10)	HIITG (n = 10)	CMT-PBMTG (n = 10)	HIIT-PBMTG (n = 9)	CG (n = 10)	p-value
**Sample characterization**						
Gender (M/F), n	8/2	7/3	4/6	7/2	7/3	0.32
Age (years)	68.70 ± 9.97*	63.10 ± 8.22	62.10 ± 11.42	54.55 ± 7.92*	64.50 ± 8.55	0.04
Ejection fraction (%)	37.70 ± 7.35	38.70 ± 4.78#	33.90 ± 7.01	31.11 ± 5.60#	34.40 ± 5.37	0.04
Body mass (kg)	73.49 ± 15.56	77.28 ± 9.49	67.13 ± 20.16	84.44 ± 12.25	74.66 ± 16.23	0.16
BMI (kg/m^2^)	26.27 ± 3.94	28.94 ± 5.53	25.53 ± 6.38	32.03 ± 6.80	27.77 ± 5.58	0.68
**Risk factors**						
Dyslipidemia, n (%)	4 (40)	6 (60)	7 (70)	7 (77.8)	4 (40)	0.32
Hypertension, n (%)	7 (70)	7 (70)	9 (90)	6 (66.7)	10 (100)	0.25
Smoking, n (%)	4 (40)	6 (60)	3 (30)	4 (44.4)	5 (50)	0.73
Overweight, n (%)	5 (50)	7 (70)	2 (20)	6 (66.7)	6 (60)	0.17
Diabetes, n (%)	1 (10)	3 (30)	2 (20)	3 (33.3)	1 (10)	0.58
Family history of CVD, n (%)	7 (70)	6 (60)	1 (10)	1 (11.1)	1 (10)	0.003
**Drug therapy**						
Anticoagulant, n (%)	10 (100)	10 (100)	8 (80)	8 (88.9)	9 (90)	0.43
Vasodilator, n (%)	6 (60)	5 (50)	7 (70)	6 (66.7)	8 (80)	0.69
ACEI, n (%)	6 (60)	6 (60)	3 (30)	2 (22.2)	4 (40)	0.32
Antiarrhythmic, n (%)	5 (50)	2 (20)	3 (30)	1 (11.1)	4 (40)	0.36
Hypolipidemic, n (%)	8 (80)	8 (80)	6 (60)	8 (88.9)	7 (70)	0.63
Digoxin, n (%)	3 (30)	0 (0)	1 (10)	1 (11.1)	1 (10)	0.27
Beta-blocker, n (%)	7 (70)	10 (100)	9 (90)	7 (77.8)	10 (100)	0.15
Diuretic, n (%)	6 (60)	9 (90)	8 (80)	6 (66.7)	7 (70)	0.58
Hypoglycemic, n (%)	1 (10)	4 (40)	2 (20)	2 (22.2)	2 (20)	0.60
**Medical conditions**						
CABG, n (%)	3 (30)	3 (30)	2 (20)	0 (0)	2 (20)	0.48
Valvular heart disease, n (%)	0 (0)	0 (0)	1 (10)	1 (11.1)	0 (0)	0.50
Angioplasty, n (%)	9 (90)	8 (80)	5 (50)	8 (88.9)	8 (80)	0.20
AMI, n (%)	7 (70)	6 (60)	6 (60)	5 (55.6)	3 (30)	0.45
CAD, n (%)	8 (80)	9 (90)	6 (60)	5 (55.6)	5 (50)	0.26
Angina, n (%)	2 (20)	1 (10)	0 (0)	0 (0)	0 (0)	0.23
Myodilated cardiomyopathy, n (%)	0 (0)	4 (40)	1 (10)	2 (22.2)	4 (40)	0.12
AF, n (%)	3 (30)	0 (0)	0 (0)	0 (0)	0 (0)	0.01
ICD, n (%)	2 (20)	0 (0)	1 (10)	0 (0)	0 (0)	0.23
Aortic aneurysm, n (%)	1 (10)	0 (0)	0 (0)	0 (0)	0 (0)	0.40
Pacemaker, n (%)	2 (20)	1 (10)	1 (10)	0 (0)	2 (20)	0.65
Arrhythmia, n (%)	3 (30)	0 (0)	1 (10)	0 (0)	1 (10)	0.16

ACEI: angiotensin-converting enzyme inhibitor; AF: atrial fibrillation; AMI: acute myocardial infarction; BMI: body mass index; CABG: coronary artery bypass graft surgery; CAD: coronary artery disease; CG: control group; CMT-PBMTG: continuous moderate-intensity training + photobiomodulation group; CMTG: continuous moderate-intensity training group; CVD: cardiovascular disease; HIIT-PBMTG: high-intensity interval training + photobiomodulation group; HIITG: high-intensity interval training group; ICD: implantable cardioverter-defibrillator.

Mean values for CPET and exercise tolerance variables across all groups are presented in Supplementary Table 1 . p-values from the GEE model effects test are available in Supplementary Table 2 . The primary results showing statistically significant differences are illustrated in Figure 1 (speed) and Figure 2 (grade). Central Illustration also outlines the main findings and illustrates the overall structure of the intervention protocol.

**Figure 1:** – Speed pre- and post-intervention for the 5 groups. CG: control group; CMTG: continuous moderate-intensity group; CMT-PBMTG: continuous moderate-intensity group plus photobiomodulation; HIITG: high-intensity interval training group; HIIT-PBMTG: high-intensity interval training group plus photobiomodulation. *indicates significant differences between pre- and post-intervention within the same group. # indicates significant differences between groups at the same time point.

**Figure 2:** – Grade pre- and post-intervention for the 5 groups. CG: control group; CMTG: continuous moderate-intensity group; CMT-PBMTG: continuous moderate-intensity group plus photobiomodulation; HIITG: high-intensity interval training group; HIIT-PBMTG: high-intensity interval training group plus photobiomodulation. *indicates significant differences between pre- and post-intervention within the same group. # indicates significant differences between groups at the same time point.

Although some numerical improvements were observed in VO_2_peak, VE/VCO_2_slope, and oxygen uptake efficiency slope, no statistically significant differences were found between groups for these variables. These findings suggest that the intervention, with or without photobiomodulation, did not produce meaningful changes in these CPET parameters.

## Discussion

To the best of our knowledge, this is the first study to investigate the effectiveness of PBMT when combined with CMT or HIIT, and whether it provides additional benefits beyond CMT or HIIT alone in improving VO_2_peak, other CPET-derived variables, and exercise tolerance in individuals with HF. The main findings are summarized below.

The primary result was that all intervention groups demonstrated significant improvements in exercise tolerance, as evidenced by increased speed and grade during the CPET. However, when comparing groups receiving PBMT with those that did not, no additional benefit of PBMT was observed for any of the analyzed variables. This finding was corroborated by post-hoc analysis, which revealed no significant differences between the PBMT and non-PBMT groups.

Our initial hypothesis posited that PBMT combined with either CMT or HIIT would yield superior outcomes compared to CMT or HIIT alone. This assumption was grounded in the mechanistic rationale that PBMT enhances mitochondrial activity by promoting the absorption of light energy by specific chromophores, thereby stimulating intracellular signaling pathways and increasing the production of ATP. These mechanisms were expected to amplify the physiological adaptations induced by AT.^20^Nevertheless, our results did not support this hypothesis, as the improvements in exercise tolerance-related outcomes were similar across all intervention groups, regardless of PBMT use. Despite this, the observed benefits underscore the clinical value of AT — particularly HIIT — in the management of HF.

Previous research in healthy individuals has shown that PBMT combined with CMT can lead to greater improvements in VO_2_peak and time to exhaustion compared to CMT alone.^12^These findings contrast with our own and may be explained by several methodological and population-related differences. For example, Miranda et al. (2018) employed a combination of laser, infrared LED, and red LED with a substantially higher total energy dose (2040 J) and 17 irradiation points, whereas our study used only laser therapy at a much lower dose (360 J) and 6 irradiation points.^12^Furthermore, while Miranda et al. studied healthy subjects undergoing only CMT, our study focused on patients with HF and included both CMT and HIIT protocols.^12^

In healthy populations, PBMT has shown positive effects on performance and fatigue-related parameters, particularly when using wavelengths of 655/950 nm (60/300 J) and a maximum power of 200 mW per diode, with application prior to exercise.^13^While these parameters partially align with those used in our study, the literature remains heterogeneous and the overall quality of evidence is low, as indicated in a meta-analysis.^12^Some studies suggest that higher doses of LLLT may be more effective, highlighting the need for further investigation — especially in clinical populations such as patients with HF, where evidence is currently limited.

Preclinical studies have demonstrated favorable effects of LLLT in rat models of HF,^16 , 21^and in healthy individuals, LLLT has been associated with improvements in functional capacity and performance indicators.^22 - 25^However, studies in cardiovascular disease populations, including those with HF, have not shown acute improvements in functional capacity when LLLT is applied prior to assessment.^14 , 15^In our study, PBMT was administered before each exercise session over a 10-week period, but no additional effects were observed, which suggests that the cumulative volume of applications alone may not be a determining factor in outcomes.

CPET parameters not only provide diagnostic and prognostic value in patients with HF, but also offer essential insights into exercise tolerance.^26^In this context, our results indicate that AT — particularly HIIT not combined with PBMT — was more effective in improving exercise tolerance parameters, such as treadmill speed and grade, compared to CMT combined with PBMT. These findings are consistent with previous studies that have reported significant benefits of HIIT alone on cardiorespiratory fitness and exercise tolerance in patients with HF.^2 , 9 - 11 , 26^

Although we did not observe statistically significant differences in VO_2_peak between groups, the absolute increases observed in all intervention groups are still noteworthy from a clinical perspective. The VO_2_peak improvements reported in our study were smaller than the 6 ml.kg^-1^.min^-1^increase reported by Wisløff et al.^11^However, the HIIT-PBMT group demonstrated a 13.5% improvement, which exceeds the 11.2% reported for HIIT alone in the study by Ulbrich et al.^27^Furthermore, the observed VO_2_peak increases in the HIIT and HIIT-PBMT groups surpass the 1 ml.kg^-1^.min^-1^threshold considered clinically relevant for maintaining autonomy and daily functional capacity.^28^

While VO_2_peak did not differ significantly across groups, the improvements in performance metrics such as speed and grade suggest possible adaptations related to movement economy. In other words, participants may have become more efficient in their movements, performing higher-intensity tasks with the same or lower metabolic cost. This type of adaptation is particularly meaningful in patients with HF, as it reflects a functional improvement without additional physiological burden.

### Strengths and limitations of the study

The present study has several strengths. Its design is applicable to clinical practice and produced reliable results regarding functional capacity using CPET-derived metrics — the gold standard for evaluating cardiorespiratory fitness. To the best of our knowledge, this is the first study to examine the effects of PBMT combined with 2 distinct AT modalities (CMT and HIIT) in patients with HF. Moreover, it appears to be the first to employ long-term PBMT as part of a Cardiac Rehabilitation Program in this population. These novel findings contribute to the growing body of evidence supporting PBMT as a potential adjunctive, nonpharmacological therapy in the management of HF, demonstrating that it is as safe and feasible as conventional AT alone.

Nonetheless, this study has important limitations. First, we did not include a group treated exclusively with PBMT, which would have allowed us to isolate its effects. Second, the sample size may have been insufficient to detect significant differences in some of the pre- and post-intervention comparisons. The absence of a placebo group undergoing the same AT protocols without active PBMT irradiation is another limitation, as is the nonrandomized study design. Additionally, this clinical trial was not registered, which limits its transparency and reproducibility. Finally, participants in the CG were those who declined to participate in the intervention program, introducing a potential selection bias that must be acknowledged.

## Conclusion

In summary, our findings indicate that PBMT, when combined with either CMT or HIIT, did not demonstrate superior efficacy in improving the evaluated outcomes compared to CMT or HIIT alone. Although PBMT has shown potential benefits in other settings, its additional value in enhancing exercise tolerance, VO_2_peak, and other CPET-derived variables in patients with HF was not substantiated in the present study.

Based on the mechanistic rationale for PBMT’s role in enhancing cellular metabolism and production of ATP, we recommend that future studies investigate alternative therapeutic parameters, such as higher energy doses, more irradiation points, or different wavelengths, to further evaluate PBMT’s potential as a nonpharmacological intervention for HF. Such research could offer valuable insights into optimizing adjunctive therapies for this population.

## *Supplemental Materials

Supplementary material 1 For additional information Supplemental Material 1, please click here. https://abccardiol.org/supplementary-material/2025/12211/2025-0086_AO_Supplementary_table_1.pdf

Supplementary material 2 For additional information Supplemental Material 2, please click here. https://abccardiol.org/supplementary-material/2025/12211/2025-0086_AO_Supplementary_table_2.pdf
